# Abnormal Default-Mode Network Homogeneity in First-Episode, Drug-Naive Major Depressive Disorder

**DOI:** 10.1371/journal.pone.0091102

**Published:** 2014-03-07

**Authors:** Wenbin Guo, Feng Liu, Jian Zhang, Zhikun Zhang, Liuyu Yu, Jianrong Liu, Huafu Chen, Changqing Xiao

**Affiliations:** 1 Mental Health Center, the First Affiliated Hospital, Guangxi Medical University, Nanning, Guangxi, China; 2 Key Laboratory for NeuroInformation of Ministry of Education, School of Life Science and Technology, University of Electronic Science and Technology of China, Chengdu, Sichuan, China; University of Maryland, College Park, United States of America

## Abstract

**Background:**

Default mode network (DMN) is one of the most commonly recognized resting-state networks in major depressive disorder (MDD). However, the homogeneity of this network in MDD is poorly understood. As such, this study was conducted to determine whether or not an abnormal network homogeneity (NH) of DMN is observed in patients with first-episode and drug-naive MDD.

**Methods:**

Twenty-four first-episode drug-naive patients with MDD and twenty-four healthy control subjects participated in the study. NH and independent component analysis (ICA) methods were used to analyze data.

**Results:**

Depressed patients exhibited a significantly increased NH in the left dorsal medial prefrontal cortex (MPFC) and decreased NH in the right inferior temporal gyrus (ITG) compared with the healthy control subjects. Receiver operating characteristic curves (ROC) were analyzed and results revealed that the NH values of MPFC and ITG could be applied as candidate markers with relatively high sensitivity and specificity to distinguish patients from healthy control subjects. No correlation was observed between the NH values of the two regions and clinical variables.

**Conclusions:**

Our findings suggested that an abnormal DMN homogeneity could be observed in MDD, which highlight the importance of the DMN in the pathophysiology of MDD.

## Introduction

As a common psychiatric disorder, major depressive disorder (MDD) is characterized by persistent and pervasive feelings of sadness, guilt, and worthlessness [1]. Despite the rapid progress in the development of antidepressants, approximately 60% of depressed patients suffer at least one recurrence [2] and the pathophysiology of MDD remains equivocal.

Default-mode network (DMN) is involved in self-referential activities [3], such as mental time travel, perspective taking, and mind theory [4], [5]. Evidence has shown that DMN plays a key role in neural activities mediating MDD [6]. DMN comprises a specific set of brain regions, including the medial prefrontal cortex (MPFC), posterior cingulate cortex/precuneus (PCC/PCu), medial parietal cortex, lateral parietal cortex, and inferior parietal cortex; these regions routinely show decreased activities during task-related cognitive processes [7]. Independent component analysis (ICA) and region-of-interest (ROI) seed-based correlation approaches are the most commonly employed approaches to assess resting-state brain networks. ICA is a model-free approach used to estimate largely overlapping spatial processes. ROI seed-based correlation method is utilized to determine the temporal coherence between the time series of a particular ROI and the time series of all other voxels in the brain by performing correlation or regression analysis [8]. Further studies have also focused on other analytical methods, such as whole brain partial correlation methodology in MDD [9].

Abnormalities in the connectivity of DMN have been associated with the pathophysiology of MDD; however, inconsistent results have been obtained. Increased functional connectivity (FC) in DMN has been found in MDD. For example, increased subgenual cingulate and thalamic FC with DMN are observed in depressed patients by ICA [10]. Sheline et al. [11], Zhou et al. [12], and Hamilton et al. [13] also reported increased FC in the DMN of depressed patients. Two spatially independent subnetworks of DMN, namely, the anterior and posterior subnetworks, have been further identified in MDD; both subnetworks exhibit an increased FC in the pre-treatment of MDD [6]. Hamilton et al. [13] further reported that the dominance level of DMN is related to the extent of maladaptive depressive rumination and adaptive reflective rumination.

Studies have also observed the decreased FC in DMN in MDD. Decreased FC between PCC/PCu and the bilateral caudate has been detected in depressed individuals [14]. Other studies have also revealed the depression-related reduced FC in DMN [15], [16]. In our previous study, voxel-mirrored homotopic connectivity (VMHC) method has been used and revealed a decreased interhemispheric homotopic connectivity in MPFC and PCC/PCu in MDD, but DMN has not been primarily investigated in this study [17]. Furthermore, a dissociation pattern of regional activity has been observed in the DMN in first-episode, drug-naive MDD at rest [18]. Interestingly, increased and decreased FC in DMN have been noted in late-life depressed patients [19] and in first-episode, drug-naive adults with MDD [20]. These findings indicated that further studies on the integrity of DMN in MDD should be conducted.

Previous studies revealed different findings regarding abnormal DMN activities. Such differences can be attributed to the heterogeneity of studies in terms of sample size, illness duration, depression severity, treatment effect, analytical methods, and study designs, such as resting-state versus task-related. These DMN alterations in MDD have also been revealed by connectivity methods, such as ICA or ROI seed-based correlation method. Although these two methods provide important information from data analysis, both methods exhibit various drawbacks. For ICA, the most efficient method that can be used to compare components among subjects and/or between groups is unclear. For the ROI seed-based correlation method, it seems to be arbitrary to place the ROI seed within a network. Therefore, new methods used to analyze functional neuroimaging data should be developed.

In the present study, a novel method termed network homogeneity (NH) was used [21] to conduct an unbiased survey of the DMN by estimating the homogeneity of the whole network. NH is a voxel-wise measure used to evaluate the correlation of a voxel with all other voxels in a particular network. In this case, homogeneity is defined as the mean correlation of any specific time series of a voxel with the time series of every other voxel in the network. This method also provides a potential alternative approach to assess the homogeneity of specific large-scale networks. In contrast to ROI seed-based correlation method and ICA used for resting-state FC, the NH approach can be used to conduct a hypothesis-driven interrogation of long-range networks related to clinical applications; in this approach, the advantages of the two previous methods are combined [21]. In clinical applications, NH method can be applied to analyze data related to attention-deficit/hyperactivity disorder (ADHD) [21] and schizophrenia [22]. On the basis of our previous findings [17], [18] and the functional associations of DMN, we hypothesized that abnormal DMN homogeneity is detected in the MDD group but not in the control group. We also hypothesized that abnormal DMN homogeneity is correlated with the clinical variables in patients with MDD.

## Methods and Materials

### Subjects

All of the participants were informed regarding the procedures and signed a written informed consent. The study was approved by the ethics committee of the First Affiliated Hospital, Guangxi Medical University, China.

Twenty-five first-episode drug-naive patients were consecutively recruited from the Mental Health Center, the First Affiliated Hospital, Guangxi Medical University, China. Twenty-five right-handed healthy control subjects were also recruited from the community. Depression attack was diagnosed according to the Structured Clinical Interview of the DSM-IV (SCID) [1]. The severity of depression was evaluated using a 17-item Hamilton rating scale for depression (HRSD) [23]. Executive function was assessed by Wisconsin card sorting test (WCST, 48 cards) [24]. The inclusion criteria of the patients were described as follows: 1) first-episode and drug-naïve conditions; 2) current episode of depression with HRSD total score ≥18; and 3) illness duration ≤1 year. The following exclusion criteria for both groups were considered: 1) other Axis I psychiatric disorders, such as schizophrenia, schizoaffective disorder, bipolar disorders, anxiety disorders, or severe Axis II personality disorders or mental retardation, assessed with SCID; and 2) a history of organic brain disorders, neurological disorders, cardiovascular diseases, or other serious physical illnesses revealed by personal history or laboratory analysis. None of the control subjects had a family history of major psychiatric or neurological illness in their first-degree relatives.

### Image Acquisition

Imaging was conducted using a Siemens 3 T scanner. The participants were instructed to keep their eyes closed, remain motionless, and stay awake. An echo-planar imaging (EPI) sequence with the following parameters was used: repetition time/echo time (TR/TE) = 2000/30 ms; 30 slices, 64×64 matrix; flip angle, 90°; FOV, 24 cm; slice thickness, 4 mm; gap, 0.4 mm; and 250 volumes (500 s).

### Data Pre-processing

Data were pre-processed in Matlab (Mathworks) by using data processing assistant for resting-state fMRI (DPARSF) [25]. After corrections for slice timing and head motion were conducted, the subjects included in the analysis should have a maximum displacement ≤2 mm in *x*, *y*, or *z* direction, and should have an angular rotation <2° on each axis. The scans were normalized to the standard SPM8 echo-planar imaging template. Two steps were involved in normalization: linear registration and non-linear registration [26], [27]. In brief, registration was initially performed by matching the whole brain with a template to determine the optimum 12-parameter affine transformation (including three translations, three rotations, three shears, and three zooms). Afterward, registration proceeded by matching the brains together with an appropriate weighting of the template voxels. A Bayesian framework was applied to maximize a posteriori probability of being correct. For instance, it maximizes the product of the likelihood function (derived from the residual squared difference) and the prior function (based on the probability of obtaining a particular set of zooms and shears). Non-linear deformations were estimated by linearly combining the three dimensional discrete cosine transform (DCT) basis functions. These parameters represented the coefficients of the deformations in three orthogonal directions. Matching was conducted with the following procedures. The bending energies of the deformation fields and the residual squared difference between images and templates were simultaneously minimized. After spatial normalization was conducted, the images were re-sampled to obtain the dimensions of 3 mm×3 mm×3 mm; temporal bandpass was then filtered (0.01 Hz to 0.08 Hz) and linearly detrended. Several sources of spurious covariates with temporal derivatives were removed from the images by linear regression; six head motion parameters obtained by rigid body correction, a signal from a ventricular ROI, and a signal from a region centered in the white matter [28] were also removed. However, the removal of the global signal in the pre-processed resting-state FC data is debatable [29], [30], [31]. Therefore, the global signal was included in the current study.

### DMN Identification

As suggested by two previous studies [18], [32], group ICA method was used to select DMN as a mask from healthy control subjects. Three main steps, including data reduction, independent component (IC) separation, and back reconstruction, were performed using toolbox GIFT (http://mialab.mrn.org/software/#gica). DMN was chosen according to the templates provided by GIFT, which was applied in NH analyses.

### NH Analyses

NH analyses were conducted using Matlab (Mathworks). For each participant, voxel-wise NH was calculated using the equation described in a previous study [21]. The average NH of each voxel in the DMN mask was obtained, and the averaged NH maps were smoothened with a Gaussian kernel of 8 mm full-width at half-maximum.

### Statistical Analyses

Demographic and clinical data were compared using two-sample *t*-test and Chi-square test when appropriate. After assessing the normality of the NH data (*p*>0.5) by software REST [33], we performed NH analyses with two-sample *t*-tests by using voxel-wise cross-subject statistics in DMN. Significant level was set at the corrected *p*<0.05 for multiple comparisons according to Gaussian random field (GRF) theory (min *z* >1.96, cluster significance: *p*<0.05). Considering that resting-state FC can be affected by micromovements from volume to volume [34], we calculated the framewise displacement (FD) values of each subject. The mean FD was used as a covariate in the group comparison of NH.

Brain regions with abnormal NH were identified as ROIs. Mean *z* values in these ROIs were extracted for further receiver operating characteristic curve (ROC) analysis and correlation analysis. In addition, the normality of the mean *z* values in these ROIs was assessed. The obtained *p* values were 0.764 and 0.984 for the left dorsal MPFC and the right inferior temporal gyrus (ITG), respectively.

## Results

### Subjects

One patient and one control subject were excluded from further analyses because of excessive head motion during image acquisition. The two groups did not differ significantly in terms of age (*t*-tests *t = *0.89, df = 46, *p* = 0.38), sex ratio (Chi-square test *x^2^* = 0.09, df = 1, *p = *0.77), and years of education (*t*-tests *t* = –1.60, df = 46, *p* = 0.12; Table 1).

**Table 1:** Demographic and clinical features of the participants.

Demographic data	MDD patients (*n* = 24)	Healthy controls (*n* = 24)	*p* value
Gender (male/female)	13/11	14/10	0.77^a^
Age (years)	25.58±7.45	24.04±4.04	0.38^b^
Years of education (years)	12.92±3.37	14.38±2.92	0.12^b^
Mean displacement (mm)	0.09±0.04	0.09±0.03	0.16^b^
HRSD score	25.75±5.86		
Illness duration (months)	4.96±3.39		
WCST			
Number of categories achieved	3.39±1.95		
Number of errors	22.00±11.29		
WCST-Pre	8.00±5.25		

a *p* value for gender distribution obtained by chi-square test.

b *p* values obtained by two samples *t*-tests.

MDD = major depressive disorder; HRSD = Hamilton Rating Scale for Depression; WCST-Pre = persistent error response of Wisconsin Card Sorting Test.

### DMN Maps Determined by ICA

**Figure 1:**
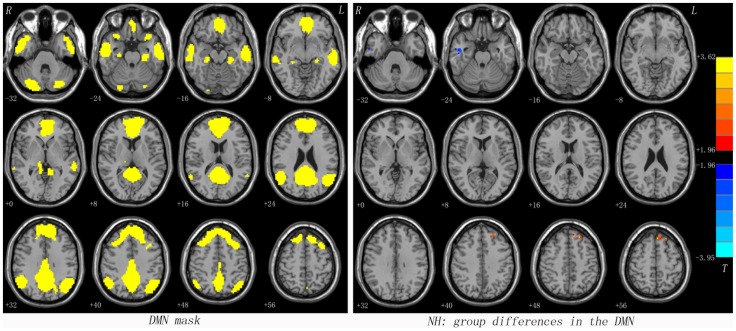
The DMN mask determined by ICA from the control group and statistical maps showing NH differences between subject groups. Red and blue denote higher and lower NH, respectively. Colored bars indicate the *T* value from two-sample *t*-tests. DMN = default mode network; ICA = independent component analysis; NH = network homogeneity.

Using a group ICA approach, we selected DMN from the control subjects. DMN was used as a mask in the following NH analyses and comprised brain areas, including bilateral MPFC, ventral anterior cingulate cortex (ACC), PCC/PCu, lateral temporal cortex, medial parietal lobe, lateral parietal lobe, inferior parietal lobe, and the cerebellar Crus I and Crus II (Figure 1).

### NH: Group Differences in the DMN

Using two-sample *t*-tests by voxel-wise cross-subject comparisons, we observed a significant group difference in NH values in DMN between the patients and the control subjects. The NH in the left dorsal MPFC was higher in the patient group than in the control group. By comparison, the NH in the right ITG was lower in the patient group than in the control group (Figure 1 and Table 2).

**Table 2:** Significant NH differences between groups.

Cluster location	Peak (MNI)			Cluster size	*T* value
	*x*	*y*	*z*		
Patients > Controls					
Left dorsal MPFC	−18	36	45	88	3.6207
Patients < Controls					
Right ITG	48	−6	−21	35	−3.1092

NH = network homogeneity; MNI = Montreal Neurological Institute; MPFC = medial prefrontal cortex; ITG = inferior temporal gyrus.

### ROC Analyses in Patients and Control Subjects

**Figure 2:**
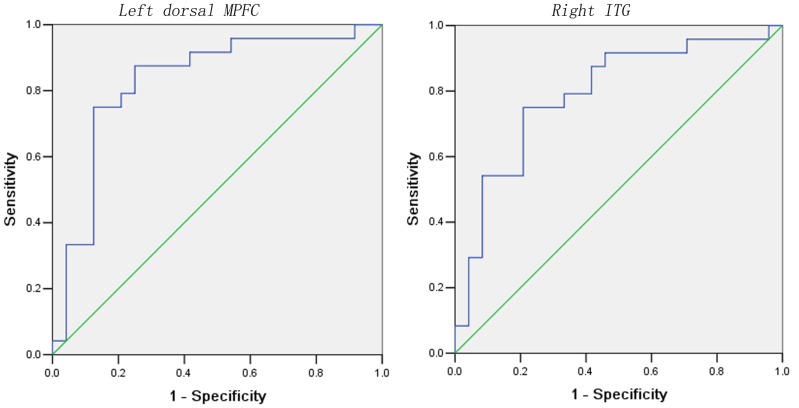
Receiver operating characteristic (ROC) curves using the mean NH in the left dorsal MPFC and the right ITG to separate the patients from healthy controls. NH = network homogeneity; MPFC = medial prefrontal cortex; ITG = inferior temporal gyrus.

Two regions (the left dorsal MPFC and the right ITG) with abnormal NH were observed in the patient group. This result indicated that the NH values of these two regions may be utilized to separate the two groups. In Figure 2 and Table 3, the areas under the curves of the two regions were high. This result was further analyzed. The results showed that the NH values of these two regions could be used as candidate markers to distinguish patients from control subjects with relatively high sensitivity and specificity.

**Table 3:** ROC analysis for differentiating patients from healthy controls.

Brain regions	Area Under the Curve	Cut-off point	Sensitivity	Specificity
Left dorsal MPFC	0.828	0.0162	75.00% (18/24)	87.50% (21/24)
Right ITG	0.790	−0.6975	79.17% (19/24)	75.00% (18/24)

ROC = receiver operating characteristic; NH = network homogeneity; MPFC = medial prefrontal cortex; ITG = inferior temporal gyrus.

### Correlations between NH and Clinical Variables

No correlation was found between the NH values of the left dorsal MPFC and the right ITG and the HRSD or WCST scores of the patient group. No correlation was also observed between NH and age, education level, or illness duration.

## Discussion

Using the NH approach, we provided an unbiased survey of the DMN in first-episode, drug-naive MDD at rest. Our principal finding was that the patients exhibited a higher NH in the left dorsal MPFC and a lower NH in the right ITG than the control subjects. Further ROC analyses suggested that the abnormal NH in the left dorsal MPFC and the right ITG could be used as candidate markers to distinguish patients with MDD from healthy control subjects.

Increased self-focus is reported as the core psychopathology of a resting state activity in MDD, which is characterized by the increased self-awareness and decreased focus on environmental phenomena, including ongoing events, persons, and objects [35]. Enhanced self-focus is attributed to negative emotions to oneself and increased cognitive processing of oneself with subsequent ruminations [36]. The dorsal MPFC is regarded as a key area in DMN and dorsal cognitive system. Therefore, we speculated that the increased NH of the left dorsal MPFC may affect the function of this region and prevent the coordination between the dorsal cognitive system and the ventral emotional system. Predominantly negative emotions and increased cognitive deficits have been observed in depressed patients because of this failure [36]. The present results are similar to those of a previous study [11], which showed an increased FC in the bilateral dorsal MPFC in the MDD group. In the same study, the dorsal MPFC connectivity is positively correlated with HRSD scores, suggesting that the functional abnormality in the dorsal MPFC corresponds to emotional dysregulation [11]. MPFC is strongly associated with affective-limbic regions (such as the amygdala and the hippocampus) and executive control as well as emotional processing regions (such as the orbital frontal cortex and the ACC) [37]. Therefore, MPFC may participate in emotional processing and self-referential processing via these links [38]. Any dysfunction in this region, such as the increased NH in the present study, is likely associated with the failure in the modulation of emotional behaviors and self-referential processes investigated with task-related and resting-state functional neuroimaging techniques [39], [40]. In other studies, MPFC exhibits high diagnostic and prognostic accuracy to separate patients with treatment-resistant depression from individuals with treatment-sensitive depression [41]. Consistent with this finding, the NH values of the left dorsal MPFC in the present study could be utilized as a candidate marker with relatively high sensitivity and specificity to differentiate depressed patients from healthy control subjects.

The ITG is one of the most consistently detected regions in the pathophysiology of MDD [42] because this region participates in emotional processing and social cognition [43]. Decreased activation and connectivity in the ITG have been observed in MDD at rest or in response to sad stimuli [42], [44]. We also observed an increased regional homogeneity (ReHo) in the right ITG in first-episode, treatment-sensitive MDD from a 1.5 T scanner [45]. We recruited similar patients and identified the decreased NH values in the right ITG in MDD in the present study. Although the results from these two studies could not be compared directly because different scanners and analyses were applied, the results from both studies indicated a functional abnormality in the ITG. Moreover, the ITG is a key node in a widespread network of frontal, temporal, parietal, occipital, and sub-cortical structures, which can be observed to provide a diagnostic and prognostic prediction to treatment responses [46]. Similar to the results of the previous study [46], the present ROC results suggested that the NH values of the right ITG could be used as a candidate marker to distinguish patients from healthy control subjects.

The present results exhibited a dissociation pattern of NH in the anterior and posterior parts of DMN, in which an increased NH was observed in the left dorsal MPFC and a decreased NH was found in the right ITG in MDD. This pattern is supported by a recent ICA study [20], which reported a dissociation between the anterior and posterior FC in the resting-state DMN in first-episode, drug-naive patients with MDD. This dissociation pattern is also observed in our previous study, in which an increased regional activity is observed in the left dorsal MPFC and a decreased regional activity is found in the left parahippocampal gyrus (PHG) by using a fractional amplitude of low-frequency fluctuations (fALFF) method [18].

Considering that clinical characteristics, such as illness duration and depression severity, are correlated with the abnormal FC of the DMN [9], [17], we predicted that abnormal DMN homogeneity was correlated with the clinical variables of patients with MDD. However, our study revealed that abnormal DMN homogeneity was not correlated with the clinical variables, and this result was unexpected. In our previous study, a significantly positive correlation was observed between VMHC in MPFC and persistent error response of WCST (WCST-Pre) in the same sample [17]. These inconsistent findings can be attributed to the different analytical methods used in the studies. In the previous study, VMHC was used to quantify the resting-state FC between each voxel in one hemisphere and its corresponding voxel in the opposite hemisphere. In the present study, NH is a voxel-wise measure that can be performed to evaluate the correlation of a voxel with all other voxels in the DMN. Different analytical methods may have also contributed to the different clusters of MPFC observed in the two studies, particularly the left dorsal MPFC in the present study and the bilateral ventral MPFC in the previous study.

Several limitations should be noted in this study. First, a relatively small sample size was used in the study. Second, the DMN mask was derived from healthy control subjects by using ICA; thus, bias in the analyses may be introduced. Third, the present study focused on DMN, and this approach may elucidate the pathophysiological contribution of DMN; however, relevant findings from other brain regions may be neglected. Finally, age, sex ratio, and education level were not used as covariates to remove the possible confounding effects as conducted in other studies [9], [47]. This procedure may yield biased results, but no statistically significant differences in these parameters were observed between patients and control subjects. Moreover, imaging studies have not applied these parameters as covariates [20], [48]. Hence, the decision to consider the confounding effects of age, sex ratio, and education level slightly affects the final results.

Despite these limitations, the present findings showed the abnormal NH of DMN in MDD. These abnormalities could be applied as candidate biomarkers to distinguish patients from healthy control subjects. Thus, the results highlighted the importance of DMN in the pathophysiology of MDD.
